# Knowledge, Attitude, Practice, and Perceived Barriers of Colorectal Cancer Screening among Family Physicians in National Guard Health Affairs, Riyadh

**DOI:** 10.1155/2014/457354

**Published:** 2014-09-28

**Authors:** Eyad Demyati

**Affiliations:** Department of Family Medicine and Primary Health Care, King Abdulaziz Medical City, National Guard Health Affairs, P.O. Box 22490, Riyadh 11426, Saudi Arabia

## Abstract

*Objectives.* The objective of this study is to explore the current knowledge, attitude, and practice of family physicians working in family medicine clinics in National Guard Health Affairs (NGHA), Riyadh, toward colorectal cancer (CRC) screening and to identify the barriers of the screening. *Methods.* Data were collected using a validated self-administered questionnaire adopted from the National Cancer Institute in USA, customized by adding and eliminating questions to be in line with the institution (NGHA) characteristics. *Results.* Of the 130 physicians, 56.2% of the physicians were not practicing CRC screening although 94.6% considered CRC screening effective. Board certified physicians had higher knowledge score and were practicing CRC screening more when compared to other physicians. Physicians who reported practicing CRC screening scored more on the knowledge score than those not practicing. Male physicians scored better on attitude score than female physicians. The study found that barriers were cited in higher rates among physicians not practicing CRC screening compared with practicing physicians. Lack of patients' awareness was the most cited barrier. *Conclusion.* Large percentage of family physicians in this study do not practice CRC screening, despite the knowledge level and the positive attitude.

## 1. Introduction

“Cancer is a leading cause of death worldwide” [1]. Colorectal cancer (CRC) ranks fourth in the most common cause of death due to cancers worldwide [2]. In USA, CRC is the second leading cause of cancer-related deaths in cancers affecting both male and female [3].

According to the Saudi Cancer Registry, CRC ranks second in the most common cancers among Saudis after breast cancer and first among Saudi males with constant rise in the incidence for the past few years. Furthermore, the overall survival rate (44.6%) is generally lower than the typically reported survival rates all over the world. CRC in Saudi Arabia present late with metastasis and obstruction in percentages more than what is reported in western communities. Nearly, 40% of CRC diagnosed were equal to or below 50 years of age with mean age of 58 years, which is lower than developed countries [4–8].

CRC is an ideal tumor for screening, with its high incidence and long time between adenomatous polyp and carcinoma, and early diagnosis by screening can reduce incidence and mortality of the disease [9–14].

Family physicians have a key role in screening practice due to frequent contact with large proportion of population. Furthermore, the involvement of family physicians in screening program implementation has been recommended by several guidelines [9].

However, physicians-reported CRC screening recommendation rates in the literature are still below the demand if a major impact on CRC mortality is targeted [15]. Moreover, this is the first local study in Saudi Arabia that is looking for data regarding CRC screening practice. The aim of this study is to explore the current knowledge, attitude and practice of family physicians toward CRC screening and to identify the barriers of conducting such a screening.

## 2. Methods

A cross-sectional descriptive study was carried out among family physicians working in family medicine clinics in National Guard Health Affairs (NGHA), Riyadh, Saudi Arabia between February and March 2013. All 170 family physicians were included in the study without sampling.

A validated questionnaire was adopted from the National Cancer Institute in USA, customized by adding and eliminating questions to be in line with the institution (NGHA) characteristics. A pilot study on 10 physicians was conducted to check understanding of the questionnaire, resulted in some vocabularies replacement and format enhancing to avoid confusion. Then the questionnaire was reviewed and approved by tow reviewers from King Abdulla International Medical Research Center (KAIMRC). It contains demographic characteristics, questions to assess the knowledge, attitude, current practice and barriers of CRC screening. Questionnaires were handed out to the physicians during working hours by the researcher or an assigned nurse and collected in the next following day. It was self-administered and takes about 10 minutes to be completed.

Data management and statistical analysis were carried out using Statistical Package for Social Sciences (SPSS) software version 20.0. Categorical variables were presented as frequencies and percentages, Chi-Square test (*χ* ^2^) used to explore the association between variables.

Knowledge score: correct answers marked with 1 and wrong answers with 0. A sum of all knowledge questions was calculated for each participant. Knowledge score was computed on the basis of 11 questionnaire items, the answers were evaluated according to the Centers for Disease Control and prevention (CDC) and United States Preventive Services Task Force (USPSTF) Guidelines recommendations.

Attitude score: Answers with positive attitude marked with 1 and negative attitude marked with 0. A sum of all attitude questions was calculated for each participant. Attitude score was computed on the basis of 8 questionnaire items.

Means of scores were compared between groups using student *t*-test (*t*). *P*-value at 0.05 or less was considered to be significant.

Permission from KAIMRC in Riyadh was obtained. Questionnaire cover sheet of the study explained that the participation of the physicians considered as a consent form of agreement.

## 3. Results

### 3.1. Demographics

The study included 170 physicians; the response rate was 76%. Out of 130 physicians, 68 (52.3%) were females, 51 (39%) were family medicine board certified, and 56 (43%) were MBBS holders (Table 1). Mean age was 38 years (SD 7.4).

### 3.2. Knowledge

The mean value results for the knowledge score was founded to be 5.02 with standard deviation of 2.73 and ranged between 0–11 (Table 2).

Board certified physicians had higher knowledge score than other physicians (*t* = 2.6; *P* = 0.009).

Physicians who reported practicing CRC screening scored more on the knowledge score than those not practicing, (*t* = 2.7; *P* = 0.007).

Physicians influenced by USPSTF Recommendations achieved better knowledge score compared to those who were not (*t* = 3.1; *P* = 0.002).

Physicians influenced by their patients' preference for CRC screening also achieved better knowledge score when compared to those who were not (*t* = 3.2; *P* = 0.002).

Although not statistically significant, male physicians had higher knowledge score than female (*t* = 1.8; *P* = 0.071) (Table 3).

### 3.3. Attitude

Among the sample studied, 123 physicians (94.6%) considered CRC screening for asymptomatic average-risk patients to be effective and 106 (81.5%) prefer having structured screening program over opportunistic screening.

Among the screening modalities, colonoscopy was cited as “very effective” by 123 (94.6%). Flexible sigmoidoscopy was considered “very effective” by 58 (44.6%) and 42 (32.3%) only for Fecal Occult Blood Testing (FOBT).

The mean value of the attitude score was found to be 6.95 with standard deviation of 1 and ranged between 4 and 8 (Table 4).

Male physicians scored better on attitude score than female (*t* = 2.3; *P* = 0.025).

Physicians aged 40 years and above had better attitude than younger physicians (*t* = 2; *P* = 0.047).

Physicians who reported having a reminder system for CRC screening scored better attitude than those who did not, (*t* = 3.4, *p* = 0.001) (Table 5).

### 3.4. Practice

Around more than half (56.2%) of the physicians are not practicing CRC screening for asymptomatic average-risk patients.

FOBT is discussed more as screening modality (80.8%) in comparison with colonoscopy (76.90%) and flexible sigmoidoscopy (35.4%). Majority of the physicians conduct FOBT by delivering stool samples to the labs (90%).

Board certification contributed significantly to practicing CRC screening when compared to other physicians (*χ* ^2^ = 7.65; *P* = 0.005).

Physicians whose practice was influenced by USPSTF and and American Cancer Society recommendations reported practicing CRC screening more than those who were not influenced by these resources (*χ* ^2^ = 4.86; *P* = 0.025) and (*χ* ^2^ = 3.92; *P* = 0.041), respectively.

Although lacking statistical significance, male physicians reported practicing CRC screening more than female physicians (*χ* ^2^ = 2.9; *P* = 0.063) (Table 6).

When asked about barriers to practice CRC screening, 80% of physicians who did not perform the screening believed time was a barrier compared to 56% of those who actually practice the CRC screening (*χ* ^2^ = 9.29; *P* = 0.002).

Also, 70% of physicians who did not practice the screening thought that the patients do not want to discuss colorectal cancer. This belief was noted among only 49% of physicians who practiced CRC screening (*χ* ^2^ = 5.78; *P* = 0.013).

Nearly, 77% of physicians who did not practice the screening reported that patients are having difficulty understanding the information about CRC screening, compared to only 45.6% of physicians who do practice the screening (*χ* ^2^ = 13.29; *P* < 0.00).

Moreover, 60.3% of physicians not practicing CRC screening believed that patients do not perceive CRC as a serious health threat, compared to only 36.8% of physicians who practice CRC screening (*χ* ^2^=7.03; *P* = 0.007).

When asked about having a clear policy and procedure for CRC screening in their workplace, 86.3% of physicians not practicing CRC screening reported that there is no policy in their workplace compared to 65% of physicians who practice CRC screening (*χ* ^2^ = 8.25; *P* = 0.004).

Also, physicians who do not practice the screening were more to report shortage of trained providers to conduct further screening procedures other than FOBT (77.8% compared to 54.4% of those who practice CRC screening) (*χ* ^2^ = 7.93; *P* = 0.004) (Table 7).

### 3.5. Perceived Barriers

Among patients related barriers for CRC screening, 92.4% of physicians reported that patients are not aware of CRC screening and (63.1%) commented that their patients have difficulties understanding the information about CRC screening.

In relation to workplace related barriers, not having a reminder system was the top barrier on the list with (86%) followed by unavailability of CRC screening policy and procedure (76.9%). Seventy percent of physicians reported that they do not have enough time to discuss CRC screening with their patients (Table 8).

## 4. Discussion

Several studies have been done in Saudi Arabia in regard to CRC, and all of them concluded and highlighted the importance of screening [4, 5, 7, 8]. This is the first study that looked at the current knowledge, attitude, practice, and perceived barriers of physicians in Saudi Arabia in order to close the gap for a better screening.

The response rate was 76%, higher than that obtained from similar studies which involved more centers and more than one city [9, 16, 17].

This study found that CRC screening is underutilized since around more than half of the study subjects were not practicing CRC screening despite a strong evidence supporting the effectiveness of the screening, physicians' knowledge, and their positive attitude toward its effectiveness, which is in accordance with findings in the literature [9, 17, 18].

Knowledge of the physicians was acceptable, and main deficiency was on the age of stopping CRC screening which is similar to Klabunde et al. findings [17].

Board certified physicians achieved higher knowledge score than physicians with lower certification, which highlights their role in educating other physicians about the importance of screening. Physicians influenced by USPSTF recommendations achieved better knowledge score compared to those who were not, and this goes in line with considering the CDC guideline as a reference for knowledge evaluation in this study and also reflects the importance of USPSTF as a trusted source of information. Physicians with a higher knowledge score were more patient-oriented in considering their patients' preference for screening, which will increase patient involvement in decision making, and that will help build a stronger doctor patient relationship. Result showed significant positive association between knowledge score and practicing CRC screening which reflects the importance of physicians' education for the improvement of CRC screening, and this finding was in accordance with the literature [19].

In general, physicians have a positive attitude toward CRC screening reflected by the attitude score. Positive attitude toward CRC screening was associated with being male; this might be due to the fact that, under the setting of NGHA clinics, female physicians are almost exclusively treating female subjects. Among female patients, other cancers (e.g., breast and cervical cancers) are of greater significance than colorectal cancer which has higher incidence among male subjects. Also, physicians aged 40 years and above had better attitude than younger physicians, which may indicate the importance of experience; there is similar finding in Greece [20]. Physicians who reported having a reminder system for CRC screening scored better attitude than those did not; this is supporting Sarfaty and Wender finding that CRC screening can be improved by implementing a reminder system targeting both doctors and patients [21]. In contrary to knowledge score, attitude score showed positive association with practicing CRC screening but the result was statistically insignificant.

Most of the physicians consider colonoscopy to be the most effective screening test, followed by flexible sigmoidoscopy. Only one-third of physicians found FOBT to be “very effective.”

In contrary, FOBT is the most used test followed by colonoscopy, which is similar to what Klabunde et al. and Federici et al. found [9, 17]. This might be due to more patients' acceptance or the availability of the FOBT in comparison to colonoscopy.

The study found that barriers were cited in higher rates among physicians not practicing CRC screening compared with practicing physicians. Lack of patients' awareness was the most cited barrier, which was also highlighted in the literature [22].

Not having a reminder system was reported by 86%. Taking that into consideration, tackling and finding solutions to overcome these barriers might improve the CRC screening.

Around 81% cited that they prefer structured screening program over opportunistic. Miles et al. concluded that the most effective way of achieving reduction in cancer related mortality is by providing screening as part of an organized program [23].

## 5. Study Limitations

1. The study was carried out in one institution in one city of Saudi Arabia and does not necessarily reflect general population.
2. Study design (cross-sectional) has limited internal validity and it is sensitive to a variety of biases.
3. Data collection tool was self-administered and lack of observation carries a risk of recalling bias or contamination by the participants.

## 6. Conclusion and Recommendations

Unfortunately, large percentage of family physicians in this study do not recommend CRC screening, despite the knowledge level and the positive attitude. This is pointing to the importance of increasing the attention of the doctors about their crucial rule in CRC prevention. Moreover, patients' involvement and awareness are of a good impact on the CRC screening process. Therefore, we recommend increasing the patients' awareness by the mass media and health campaigns and considering a national organized screening program that will help the physicians to achieve a desirable outcome.

## Figures and Tables

**Table 1:** Medical degree of the participated physicians.

Medical degree	Number of physicians	%
Family medicine board	51	39
Other		
MBBS	56	43
MRCGP	23	18
Total	**130**	**100**

**Table 2:** Knowledge score items and numbers of correct answers.

Knowledge item	Correct answers *N* (%)
Fifty years of age as starting age for screening	102 (78.5)
Awareness about stopping age for screening	51 (39.2)
Seventy-five years of age as stopping age for screening	19 (14.6)
Awareness about Fecal Occult Blood Testing	
(FOBT):	
Guaiac FOBT	68 (52.3)
Fecal immunochemical testing	46 (35.4)
FOBT office card	60 (46.2)
FOBT home kit	53 (40.8)
Ordering 3 samples for each FOBT	68 (52.7)
Screening interval for each screening modality	
FBOT annually	69 (53.1)
Flexible Sigmoidoscopy every 5 years	59 (45.4)
Colonoscopy every 10 years	57 (43.8)

**Table 3:** Knowledge score compared with different physicians' factors.

Factors	Knowledge score		
	Mean (SD)	*t*	*P*
Gender			
Male	5.47 (2.8)	1.82	0.071
Female	4.60 (2.6)		
Age			
<40 years	5.25 (2.4)	1.17	0.245
>40 years	4.68 (3.1)		
Family medicine board			
Yes	5.78 (2.8)	2.64	0.009
No	4.52 (2.6)		
Performing CRC screening			
Yes	5.74 (2.7)	2.73	0.007
No	4.45 (2.6)		
Having a reminder system			
Yes	6.18 (2.9)	1.25	0.215
No	5.29 (2.6)		
Influenced by USPSTF			
Yes	5.28 (2.7)	3.12	0.002
No	2.85 (1.9)		
Influenced by American Cancer Society			
Yes	5.12 (2.7)	1.23	0.220
No	4.20 (2.9)		
Influenced by colleagues practice			
Yes	4.76 (2.7)	1.53	0.127
No	5.53 (2.8)		
Influenced by patient's preference of screening test			
Yes	5.45 (2.8)	3.15	0.002
No	3.97 (2.2)		

**Table 4:** Attitude score items and numbers of physicians with positive attitude.

Attitude items	Positive attitude *N* (%)
CRC screening is effective for asymptomatic average risk patient	123 (94.6)
FOBT is effective	123 (94.6)
Flexible sigmoidoscopy is effective	123 (94.6)
Colonoscopy is effective	128 (98.5)
Double-contrast barium enema is effective	90 (69.2)
CT-colonography	91 (70)
Preferring structured screening program over opportunistic	106 (81.5)
Colonoscopy is the best available screening test	120 (92.3)

**Table 5:** Attitude score compared with different physicians' factors.

Factor	Attitude score		
	Mean (SD)	*t*	*P*
Gender			
Male	7.18 (0.9)	2.27	0.025
Female	6.75 (1.2)		
Age			
<40 years	6.81 (1.2)	2.00	0.047
>40 years	7.17 (0.9)		
Family medicine board			
Yes	7.12 (1.1)	1.38	0.170
No	6.85 (1.1)		
Performing CRC screening			
Yes	7.09 (1.0)	1.24	0.218
No	6.85 (1.1)		
Having a reminder system			
Yes	7.59 (0.5)	3.44	0.001
No	7.00 (1.1)		
Influenced by USPSTF			
Yes	7.00 (1.1)	1.21	0.230
No	6.62 (1.3)		
Influenced by American Cancer Society			
Yes	7.03 (1.0)	2.12	0.036
No	6.40 (1.4)		
Influenced by availability of providers for test other than FOBT			
Yes	6.97 (1.1)	0.48	0.631
No	6.84 (1.2)		
Influenced by colleagues practice			
Yes	6.99 (1.1)	1.53	0.609
No	6.88 (1.2)		
Influenced by patients' preference of screening test			
Yes	7.03 (1.0)	1.28	0.202
No	6.76 (1.2)		

**Table 6:** Practice compared with different physicians' factors.

Factors	Practicing CRC screening		
	%	*χ* ^2^	*P*
Gender			
Male	56.1	2.904	0.063
Female	43.9		
Age			
<40 years	64.9	1.357	0.162
>40 years	35.1		
Family medicine board			
Yes	52.6	7.646	0.005
No	47.4		
Considering screening effective			
Yes	98.2	2.626	0.107
No	1.8		
Influenced by USPSTF			
Yes	96.5	4.863	0.025
No	3.5		
Influenced by American Cancer Society			
Yes	94.7	3.916	0.041
No	5.3		
Influenced by availability of providers for tests other than FOBT			
Yes	82.5	0.698	0.278
No	17.5		
Influenced by colleagues' practice			
Yes	68.4	0.103	0.448
No	31.6		
Influenced by patient's preference of screening test			
Yes	77.2	2.025	0.109
No	22.8		

**Table 7:** Barriers among practicing physicians compared with nonpracticing physicians.

Barriers	%	*χ* ^2^	*P*
Time			
Practicing	56	9.285	0.002
Nonpracticing	80		
My patients do not want to discuss CRC screening=			
Practicing	49	5.775	0.013
Nonpracticing	70		
My patients have difficulty understanding the information about CRC screening			
Practicing	45.6	13.291	0.000
Nonpracticing	76.7		
My patients are unaware of CRC screening			
Practicing	91.2	0.167	0.465
Nonpracticing	93.2		
My patients do not perceive CRC as a serious health threat			
Practicing	36.8	7.030	0.007
Nonpracticing	60.3		
There is no CRC screening policy or procedure in my workplace			
Practicing	65	8.249	0.004
Nonpracticing	86.3		
There is no reminder system in my workplace			
Practicing	86	0.003	0.576
Nonpracticing	86.3		
Patients are not followed up through to complete CRC screening			
Practicing	68.4	3.187	0.058
Nonpracticing	82		
Shortage in trained providers for endoscopic tests as a first test			
Practicing	54.4	7.928	0.004
Nonpracticing	77.8		
Shortage in trained providers for endoscopic tests as a follow-up test			
Practicing	58	1.048	0.200
Nonpracticing	66.7		

**Table 8:** Perceived barriers reported by the physicians.

Barriers	No.	%
Patients related		
Patients are not aware of CRC screening (*n* = 130)	120	92.4
Patients do not want to discuss CRC screening (*n* = 130)	79	60.7
Patients have difficulty understanding the information about CRC screening (*n* = 130)	82	63.1
Patients do not perceive CRC screening as a serious health threat (*n* = 130)	65	50
Workplace related		
Not having a reminder system (*n* = 130)	112	86.1
Unavailability of CRC screening policy and procedure (*n* = 130)	100	76.9
Patients are not followed up through to complete CRC Screening (*n* = 129)	98	76
Shortage in trained providers for endoscopic as a first test (*n* = 129)	87	67.5
Shortage in trained providers for endoscopic as a follow-up test (*n* = 129)	81	62.8
Not having enough time to discuss CRC screening (*n* = 130)	91	70
